# Strength in Numbers:  Three Separate Studies Link in Utero Organophosphate Pesticide Exposure and Cognitive Development

**DOI:** 10.1289/ehp.1104137

**Published:** 2011-08-01

**Authors:** Kimberly Gray, Cindy P. Lawler

**Affiliations:** Division of Extramural Research and Training, National Institute of Environmental Health Sciences, National Institutes of Health, Department of Health an Human Services, Research Triangle Park, North Carolina, E-mail: Gray6@niehs.nih.gov

Children entering classrooms for the first time this fall can be seen carrying backpacks filled with school supplies, yet each new student brings something less obvious but more important—a legacy of their earlier experiences and environment. Three studies published in this issue of *Environmental Health Perspectives* (Bouchard et al. 2011; Engel et al. 2011; Rauh et al. 2011) deliver compelling new data linking one aspect of a child’s history—*in utero* exposure to organophosphates (OP), a commonly used class of pesticides—and early cognitive development. Working memory, perceptual reasoning, and IQ were among the measures of intellectual development associated with prenatal OP exposure. Bouchard et al. (2011) found that children in the category corresponding to the highest 20% of maternal urinary levels of OP metabolites during pregnancy showed a 7-point decrease in full scale IQ compared with children of mothers in the lowest 20%, an association of magnitude similar to that observed with an increase in blood lead concentrations from 1 to 10 µg/dL (Canfield et al. 2003).

The strength of evidence arising from these studies reflects, in part, their rigorous study design. Each used a longitudinal birth cohort, a design well suited to detect the evolution of exposure effects over time. Accordingly, the latest findings build on previous data from these birth cohorts showing relationships between prenatal OP pesticide exposure levels and developmental end points in the first 3 years of life (e.g., Engel et al. 2007; Eskenazi et al. 2004; Rauh et al. 2006).

Decrements in full-scale IQ, working memory, and perceptual reasoning showed the most consistent associations with prenatal exposure biomarkers across studies. Comparability of these results, despite differences in populations and exposure metrics, underscores the robustness of this latest group of findings. Two of the birth cohorts represent multiethnic inner city populations in New York City, while the third cohort comprises low-income families living in California’s Salinas Valley, an agricultural area. Rauh et al. (2011) measured chlorpyrifos in cord blood, whereas Engel et al. (2011) and Bouchard et al. (2011) relied on measurement of nonspecific OP metabolites in maternal urine samples collected during pregnancy.

A primary limitation of these three studies relates to exposure assessment. One study measured chlorpyrifos and diazinon in blood (Rauh et al. 2011), but the other studies (Bouchard et al. 2011; Engel et al. 2011) measured OP pesticide breakdown products in urine. Some of these breakdown products can also be found pre-formed in food; thus, measurements in urine do not show how much exposure was due to the parent pesticide compounds or the preformed breakdown product. Another complication is that measurement of the breakdown products reflects exposure to many different OP pesticides, some that are much more toxic than others. For many OP pesticides, however, there are no laboratory methods to measure the parent pesticides in blood or specific breakdown products in urine at levels needed for epidemiologic research. The National Institute of Environmental Health Sciences (NIEHS), through the Exposure Biology Program, is supporting efforts to improve exposure assessment for epidemiological research.

The three birth cohort studies highlighted here were initiated in the first phase of the Children’s Centers for Environmental Health and Disease Prevention Research Program. This program was developed jointly by the NIEHS and the U.S. Environmental Protection Agency (EPA) in response to a 1997 Executive Order that charged agencies to prioritize the identification and assessment of environmental health risks to children and to ensure that policies, programs, activities, and standards address these risks (Clinton 1997). At present, six full centers and six developmental/formative Children’s Centers, each comprised of interdisciplinary research teams, receive support through this program. Collectively, these centers are addressing a range of established and emerging priority issues in the environmental health of children, including neurodevelopment, respiratory health, and endocrine function.

An important component of these NIEHS/U.S. EPA Children’s Centers is meaningful engagement of affected communities. Although not the focus of the articles in this issue of *EHP*, all three research teams have community outreach and translation cores whose primary objective is to rapidly and effectively translate the center study findings at local, state, and national levels to communities and stakeholders. These efforts have taken various forms, from hosting town hall meetings, developing community advisory boards, and encouraging integrated pest management solutions in New York City to outreach efforts to reduce take home exposures among farm workers in the Salinas Valley.

The present findings in young children are likely to have implications for longer-term learning and academic success. Determining the persistence of these associations, and their generalizability to other populations, requires additional study. The U.S. EPA began a phase-out of the residential use of chlorpyrifos and diazinon in 2001 and 2004, respectively, which led to a marked reduction of markers of chlorpyrifos exposure in the United States, yet exposure to children continues due to use of organophosphate pesticides on crops and residues on foods.

Spurred by recent advances in mechanistic toxicology and exposure science, efforts to improve identification of neurodevelopmental toxicity of new and existing industrial compounds are under way (e.g., U.S. Senate Committee on Environment & Public Works 2009). The findings of Bouchard et al. (2011), Engel et al. (2011), and Rauh et al. (2011) should stimulate renewed efforts to develop and fully implement measures that can identify threats before widespread exposure occurs and harms pregnant women and children.
